# Role of Endoplasmic Reticulum Stress in Otitis Media

**DOI:** 10.3389/fgene.2020.00495

**Published:** 2020-05-27

**Authors:** Hongchun Zhao, Yanfei Wang, Bo Li, Tihua Zheng, Xiuzhen Liu, Bo Hua Hu, Juan Che, Tong Zhao, Jun Chen, Maria Hatzoglou, Xiaolin Zhang, Zhaomin Fan, Qingyin Zheng

**Affiliations:** ^1^Department of Otolaryngology-Head and Neck Surgery, Shandong Provincial ENT Hospital, Cheeloo College of Medicine, Shandong University, Jinan, China; ^2^Department of Otolaryngology/Head and Neck Surgery, Institute of Otolaryngology, Affiliated Hospital of Binzhou Medical University, Binzhou, China; ^3^Hearing and Speech Rehabilitation Institute, College of Special Education, Binzhou Medical University, Yantai, China; ^4^Clinical Laboratory, Affiliated Hospital of Binzhou Medical University, Binzhou, China; ^5^Center for Hearing and Deafness, University at Buffalo, Buffalo, NY, United States; ^6^Department of Genetics, Case Western Reserve University, Cleveland, OH, United States; ^7^Department of Otolaryngology-Head & Neck Surgery, Case Western Reserve University, Cleveland, OH, United States

**Keywords:** endoplasmic reticulum (ER) stress, streptococcal peptidoglycan polysaccharide, otitis media, tauroursodeoxycholic acid, apoptosis, therapy

## Abstract

Endoplasmic reticulum (ER) stress occurs in many inflammatory responses. Here, we investigated the role of ER stress and its associated apoptosis in otitis media (OM) to elucidate the mechanisms of OM and the signaling crosstalk between ER stress and other cell damage pathways, including inflammatory cytokines and apoptosis. We examined the expression of inflammatory cytokine- and ER stress-related genes by qRT-PCR, Western blotting, and immunohistochemistry (IHC) in the middle ear of C57BL/6J mice after challenge with peptidoglycan polysaccharide (PGPS), an agent inducing OM. We also evaluated the effect of the suppression of ER stress with tauroursodeoxycholic acid (TUDCA), an ER stress inhibitor. The study revealed the upregulation of ER stress- and apoptosis-related gene expression after the PGPS treatment, specifically ATF6, CHOP, BIP, caspase-12, and caspase-3. TUDCA treatment of PGPS-treated mice decreased OM; reduced the expression of CHOP, BIP, and caspase 3; and significantly decreased the proinflammatory gene expression of tumor necrosis factor-α (TNF-α) and interleukin-6 (IL-6). These results suggest that PGPS triggers ER stress and downstream proinflammatory gene expression in OM and that inhibition of ER stress alleviates OM. We propose that ER stress plays a critical role in inflammation and cell death, leading to the development of OM and points to ER stress inhibition as a potential therapeutic approach for the prevention of OM.

## Introduction

Otitis media (OM) is an inflammatory disorder of the middle ear associated with infection. OM is the second most common childhood disease (Hang and Brietzke, 2012). The pathogenesis of OM is related to the anatomy and immune function of patients (Gould and Matz, 2010). Both bacteria and viruses can cause an infection. Antibiotics are often recommended for infants and children with severe symptoms, such as moderate to severe ear pain and high fever, but the treatment may cause side effects such as diarrhea, vomiting, and skin rash (Han et al., 2009; Kawai and Akira, 2010; Komori et al., 2011). Antibiotic resistance remains a major public health challenge (Venekamp et al., 2013). Therefore, there is an urgent need to understand the mechanisms of OM, which reveal novel therapeutic targets for the early intervention and prevention of OM.

The host immune response has been shown to play an important role in OM pathogenesis, but the molecular details of the response are not clear. The endoplasmic reticulum (ER) is an intracellular organelle that is essential for protein folding. Errors in protein folding can lead to protein accumulation in the ER and induce the expression of ER stress-related genes, a reaction known as the unfolded protein response (UPR) (Wang and Kaufman, 2016). The signaling pathway activated by ER stress has been studied extensively, and emerging evidence has shown the contribution of ER stress to the pathogenesis of many human diseases. Recently, signaling crosstalk between ER stress and inflammatory reactions has been reported in various inflammatory disorders (Zhang et al., 2006; Zhang and Kaufman, 2008; Hasnain et al., 2012; Qiu et al., 2013; Wang and Kaufman, 2014), especially in inflammatory bowel disease (Cao et al., 2013). Previous studies suggest that inflammatory stimuli can activate the UPR, trigger ER stress, and thus damage ER homeostasis, which leads to inflammatory diseases (Zhang et al., 2006; Menu et al., 2012). However, it is also well known that inflammatory responses can induce ER stress due to overproduction of proteins in the ER (Hasnain et al., 2012). In addition to the complexity of the interplay between ER stress and inflammation, the cellular response to ER stress has cell-type-specific components that determine either adaptation to stress conditions or maladaptation and death (Guan et al., 2017). The inflammatory gene expression programs are activated during the maladaptive response to ER stress. In fact, we have shown that such a maladaptive hyperactivation of a proinflammatory response occurs during increased intensity of an environmental stress via the actions of a novel axis of the eIF2a kinase PKR interacting with the protein PACT (Farabaugh et al., 2020). Similarly, a PACT-PKR activation mechanism during ER stress has been reported to be proapoptotic (Singh et al., 2009). Therefore, data from the recent literature suggest that ER stress can lead to proinflammatory conditions or proinflammatory conditions can cause ER stress. The role of ER stress in the pathogenesis of OM is not known. We therefore tested in an experimental mouse model of OM the presence of ER stress and inflammatory gene expression and how inhibition of ER stress can affect the proinflammatory response and the pathogenesis of OM.

As demonstrated in our previous study, inoculation with the bacterial cell wall component peptidoglycan polysaccharide (PGPS) induces inflammation in the middle ear cavity, which creates an OM model without the need for live bacteria or viruses (Zhang et al., 2015). PGPS is a pathogen-associated microbial pattern (PAMP) that can activate Toll-like receptor 2 (TLR2), leading to the activation of the immune response (Han et al., 2009; Kawai and Akira, 2010; Komori et al., 2011). We previously used C57BL/6J (B6) mice with PGPS-induced middle ear inflammation as a model to investigate the pathogenesis of OM (Zhang et al., 2015). In this study, we aimed to evaluate the role of the ER stress-related inflammatory response and cell death in the development of PGPS-induced OM. We found that PGPS-induced OM is accompanied by the increased expression of inflammation-related cytokines and the expression of ER stress-related genes. We used an ER stress inhibitor, tauroursodeoxycholic acid (TUDCA), a synthetic compound that has been widely used in the treatment of OM. Interestingly, TUDCA treatment significantly alleviated middle ear inflammation and suppressed middle ear cell death. Additionally, TUDCA treatment inhibited the expression of ER stress-associated gene expression. These findings suggest that ER stress plays a key role in OM.

## Materials and Methods

### Mice and Treatments

The mice were originally obtained from the Jackson Laboratory (Bar Harbor, ME, United States). The experimental protocol was approved by the Animal Use and Care Committee of the Binzhou Medical University Hospital. A total of 168 C57BL/6JB6 mice, 8 weeks old, were used in this study. The mice were randomly assigned into three groups matched by sex and age: a PGPS-treated, a TUDCA-treated, and a PBS-treated (control) group. The PGPS-treated group was treated with PGPS (100P, BD Bioscience, San Jose, CA, United States) that was freshly prepared in 10 μl of PBS (5.5 mg/ml). This dose was selected because it balances the safety and effectiveness of the treatment, as demonstrated in our preliminary experiments (Zhang et al., 2015). The drug was injected through the tympanic membrane into the right middle ear cavity using a Hamilton syringe. The TUDCA group was treated with TUDCA (EMD Chemicals Inc., catalog no. 580549) that was freshly prepared (200 μg in 10 μl PGPS) and injected into the middle ear. The control group was treated with 0.1 M PBS. Individual mice were anesthetized intraperitoneally with 4% chloral hydrate (0.01 ml/g). Their tympanic membranes were examined at 1 h after the injection using a MedRx VetScope System^®^ otoscopic digital imaging system (MedRx, Largo, FL, United States). The mice were examined every 12 h for 3 days post-injection.

The effects of the drug treatment were evaluated by auditory-evoked brainstem response (ABR), tympanometry, histology, and expression levels of inflammation- and apoptosis-related genes.

### ABR and Tympanometry

A computer-aided evoked potential system (Intelligent Hearing Systems, Smart-EP software) and an automatic MAICO Race Car Tympanometer (MAICO Diagnostics Inc., Eden Prairie, MN, United States) were used to determine ABR thresholds and the middle ear function, respectively. The ABR and tympanometry were performed before and at 3 days post-injection with PGPS, PBS, or PGPS + TUDCA, as described previously (Zheng et al., 2007). Before entering the group, all mice had normal hearing, and electric otoscope showed normal structure without effusion and inflammation. There were no differences found between the animals in the three groups (PBS, PGPS, and PGPS + TUDCA) before injection. Briefly, mice were anesthetized with intraperitoneal injection of 4% chloral hydrate (0.01 ml/g), and their body temperatures were maintained at 37–38°C. The testing was performed in a room with environmental noise < 50 dB SPL. The stimuli included clicks and tone bursts at 8, 16, and 32 kHz. ABRs were recorded using a computer-aided evoked potential system (Intelligent Hearing Systems, Miami, FL, United States). The threshold was defined as the lowest intensity (±5 dB) at which a visible ABR wave was seen because the minimum stimulus intensity produced an ABR wave pattern similar to that of the higher-intensity stimulus (110 dB). As previously reported, young, untreated control mice showing ABR thresholds greater than 55-dB SPL (for the click stimulus), 40-dB SPL (for 8 kHz), 35-dB SPL (for 16 kHz), or 60-dB SPL (for 32 kHz) were considered hearing impaired (Han et al., 2013). Tympanogram curves were classified as type A (normal), type B (flat, clearly abnormal), and type C (indicating a negative pressure in the middle ear, possibly indicative of pathology). The results of these tests help to determine whether the eardrum is punctured, whether the fluid is present in the ear, and whether the middle ear system is working properly.

### Histological Analysis of the Middle Ear

Histological analyses were performed using the methods described previously (Han et al., 2009). On day 3 post-PGPS injection, the experimental C57BL/6J mice were euthanized with CO_2_, and their bullae (including both the middle and inner ear) were collected. Then, the bulla tissues were fixed with 10% paraformaldehyde for 24 h, decalcified with 10% EDTA solution for 5 days, and embedded in paraffin. The tissue sections (5 μm) were stained with hematoxylin–eosin (H&E) and examined under a light microscope (Leica DMI4000 B).

### Immunohistochemistry (IHC) Protocol

The tissue sections (5 μm) were deparaffinized as follows: two 10-min incubations in xylene followed by incubation in a graded series of ethanol (100, 95, 80, 70, and 50%) and then with ddH_2_O, twice, for 5 min each. The endogenous peroxidase activity was blocked at room temperature by a 20-min incubation in 3% H_2_O_2_ in PBS (pH 7.4). The sections were rinsed with PBS for 5 min and blocked in normal goat serum for 20 min at room temperature. After incubation, the residual fluid was removed (without washing). Subsequently, the sections were incubated in primary antibodies against TNF-α (Proteintech, 60291-1-Ig) and CHOP (Proteintech, 15204-1-AP) for 60 min at room temperature or 4°C overnight then rinsed twice for 5 min each. An array slide was incubated with a biotin-conjugated secondary antibody at 20–37°C for 20 min. The sections were rinsed twice for 5 min each and developed using a DAB kit (control, the degree of staining with regular microscopy) to develop. The sections were subsequently washed in distilled water, stained, and differentiated in hematoxylin. Finally, slides were dehydrated and mounted.

### Terminal Deoxynucleotidyl Transferase dUTP Nick-End Labeling (TUNEL) Staining

Apoptotic middle ear epithelial cells were identified by a TUNEL assay according to the manufacturer’s protocol. Briefly, tissue sections (5 μm) were deparaffinized and then fixed in 4% paraformaldehyde diluted in 1 × PBS (pH 7.4) for 1 h. The surface preparations were permeabilized in 0.1% Triton X-100. The specimens were stained using a TUNEL kit (*In Situ* Cell Death Detection kit, Fluorescein, Roche) at 37°C for 1 h in a humid chamber and then counterstained with DAPI for 5 min at room temperature. The tissues were then observed under a fluorescence microscope (Leica TCS SP2).

### Real-Time Quantitative PCR

The mice were sacrificed, and their right bullae (including the middle ear and inner ear) were quickly isolated. Total RNA was isolated from individual right bullae using TRIzol^®^ reagent (Invitrogen, Carlsbad, United States) according to the manufacturer’s protocol. The concentrations of RNA were measured using a Biophotometer (Eppendorf, Hamburg, Germany). The total RNA in each sample (1 μg) was reverse transcribed into cDNA using random primers following the First-Strand Synthesis protocol (Takara Bio). Quantitative real-time PCR was performed using a FastStart Universal SYBR Green Master kit (Roche, Mannheim, Germany) in a Bio-Rad iCycler iQ5 Peltier thermal cycler. The PCR thermal cycling conditions were as follows: 95°C for 10 min, 40 cycles of 95°C for 15 s, and 60°C for 1 min. Finally, a dissociation curve of 95°C for 15 s, 60°C for 1 min, 95°C for 15 s, and 60°C for 15 s was added. Primer sequences for a total of 10 genes were synthesized using Sangon Biotech Co., Ltd. (Shanghai) (see Table 1 for the gene list and primer sequences). The levels of mRNA transcripts of target genes relative to the control of GAPDH were calculated using the 2^–ΔΔCt^ method (Livak and Schmittgen, 2001).

**TABLE 1:** Primers used for Real-time quantitative PCR.

Gene	Forward primer	Reserve primer
IL-1β	gaaatgccaccttttgacagtg	tggatgctctcatcaggacag
TLR2	ctcttcagcaaacgctgttct	ggcgtctccctctattgtattg
Cas3	cggagcagtcctacatggaga	tccgtgatgtgtcgttcagat
IL-6	ggtgccctgccagtattctc	ggctcccaacacaggatga
TNF-α	gcggccacagaaaacactc	ctcccaatggtcaaggcatc
BiP	acttggggaccacctattcct	atcgccaatcagacgctcc
CHOP	ctggaagcctggtatgaggat	cagggtcaagagtagtgaaggt
Cas12	agacagagttaatgcagtttgct	ttcaccccacagattccttcc
ATF-6	tcgccttttagtccggttctt	ggctccataggtctgactcc
GAPDH	aggtcggtgtgaacggatttg	tgtagaccatgtagttgaggtca

### Western Blot

To further investigate the expression of inflammation- and ER stress-associated genes in OM, we examined protein expression using Western blot. The right bullae (including the middle ear and inner ear) were harvested immediately after euthanasia. The bullae were lysed using ice-cold RIPA buffer with protease inhibitors (cOmplete, EDTA-free cocktail tablet, Roche) and phosphatase inhibitors (PhosSTOP tablet, Roche). Lysates were incubated for 20 min on ice and then centrifuged for 30 min at 14,000 rpm at 4°C. Then, equal amounts of proteins were subjected to SDS-PAGE and transferred to a polyvinylidene difluoride (PVDF) membrane. The PVDF membrane was blocked for 3 h in 5% skimmed milk and incubated overnight at 4°C with 1:1000 diluted primary antibodies: anti-TNF-α (Proteintech, 60291-1-Ig), anti-IL-6 (Proteintech, 66146-1-Ig), anti-IL-1β (Proteintech, 60291-1-Ig), anti-GRP78/BIP (Proteintech, 11587-1-AP), anti-caspase-3 (Proteintech, 19677-1-AP), anti-caspase-12 (Proteintech, 55238-1-Ig), anti-CHOP (Proteintech, 15204-1-AP), anti-activating transcription factor 6 (ATF6; Proteintech, 24169-1-AP), and anti-GAPDH (Hangzhou Goodhere Biotechnology Co., Ltd.). All antibodies were validated for their specificity. After washing with TBST, the membrane was incubated in 1:5000 diluted secondary antibodies (Abcam, 6721, 6789). The protein bands were visualized using ECL chemiluminescence. Band intensities were quantified using ImageJ software.

### Oxidative Stress Detection

At the day 3 post-PGPS inoculation and TUDCA treatment, the mice were sacrificed, and their right bullae were dissected and placed in 10% FBS RPMI 1640 medium in a plastic culture dish. After all non-middle ear-associated tissues were removed, the mucosa in the middle ear cavity was gently removed with fine forceps and transferred to the center of a 35-mm glass-bottom culture dish and washed three times with 1 × PBS. The samples were fixed in 4% paraformaldehyde in PBS for 15 min at room temperature. Samples were washed twice in PBS to remove residual paraformaldehyde, permeabilized with 0.5% Triton X-100 in PBS for 15 min, and then washed three times in 1 × PBS. The specimens were incubated with a CellROX^®^ Green Reagent kit (Invitrogen, 10444) at 37°C for 30 min, washed three times in 1 × PBS, and then counterstained with DAPI for 5 min at room temperature. The stained tissues were observed under an immunofluorescence microscope (Leica DFC500, Germany).

### Statistical Analysis

Data are presented as the mean ± 95% confidence intervals. Statistical analyses were performed using SPSS 13.0 software. Group differences were analyzed using unpaired Student’s *t* test or one-way ANOVA (for further multiple comparison between each column, Benjamini test was applied). *P* values less than 0.05 were considered significant.

## Results

### Inflammation Presents in the Middle Ear After Inoculation With PGPS

**FIGURE 1:**
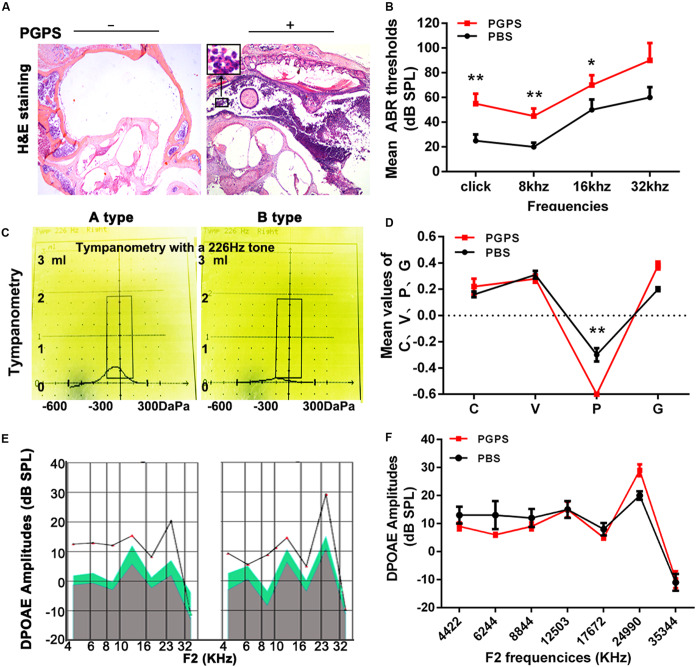
Inoculation with PGPS induces inflammatory cell infiltration in the middle ear cavity. **(A)** Histopathological analysis (H&E staining). After PGPS inoculation, inflammation became increasingly obvious as compared to the control mice (left panel, control; right panel, PGPS). The inset in the image of the PGPS-treated tissue shows the accumulation of neutrophils. **(B)** Average ABR thresholds. The mean ABR thresholds for low-frequency stimuli (clicks and 8-kHz tone bursts) and high-frequency stimuli (16-kHz tone bursts) were significantly higher in the PGPS group than in the PBS group (data = mean ± 95% CI, *n* = 10 per group per time point). **(C,D)** Quantitative analysis of average tympanometry values. Tympanogram, A type – normal; B type – flat, PGPS group. V: the mean volume value; C: compliance in tympanometry parameters; G: the gradient; P: the pressure. The negative pressure of the middle ear in the PGPS group was significantly higher than that in the PBS group (data = mean ± 95% CI, *n* = 10 per group per time point). **(E,F)** The average DP amplitudes (in dB SPL) (data = mean ± 95% CI, *n* = 10 per group per time point). Individual groups of mice from separate experiments (*P < 0.05, **P < 0.01; two-tailed Student’s *t* test).

Our previous study demonstrated that PGPS could cause inflammation of the middle ear (Zhang et al., 2015). Here, we treated the middle ear with PGPS (55 μg/10 μl) to induce OM. The PBS-treated control group had no difference in ABR thresholds and tympanometric results compared to naïve B6 mice at 8 weeks of age. Therefore, we used the PBS group as the normal control group. Mice injected with PGPS displayed greater inflammatory infiltrates in the tympanic cavity and tissue damage than the mice injected with PBS, as assessed by H&E staining (Figure 1A). Moreover, compared with PBS-injected mice, the PGPS-injected mice exhibited threshold shifts, as revealed by the ABR test (Figure 1B).

Tympanometry was used to determine the middle ear function. Most PGPS-treated mice showed a type B tympanogram, suggesting the presence of middle ear fluid and/or eardrum perforation (Figures 1C,D). However, the average DPOAE amplitude (in dB SPL) was not significantly decreased in the distortion product otoacoustic emission (DPOAE) test (Figures 1E,F). Thus, PGPS did not cause inner ear damage. These findings suggest that PGPS injection induces an inflammatory response in the middle ear cavity. We, therefore, used this model to study the effects of OM in subsequent analyses.

### PGPS-Induced Inflammatory Response and Apoptosis

**FIGURE 2:**
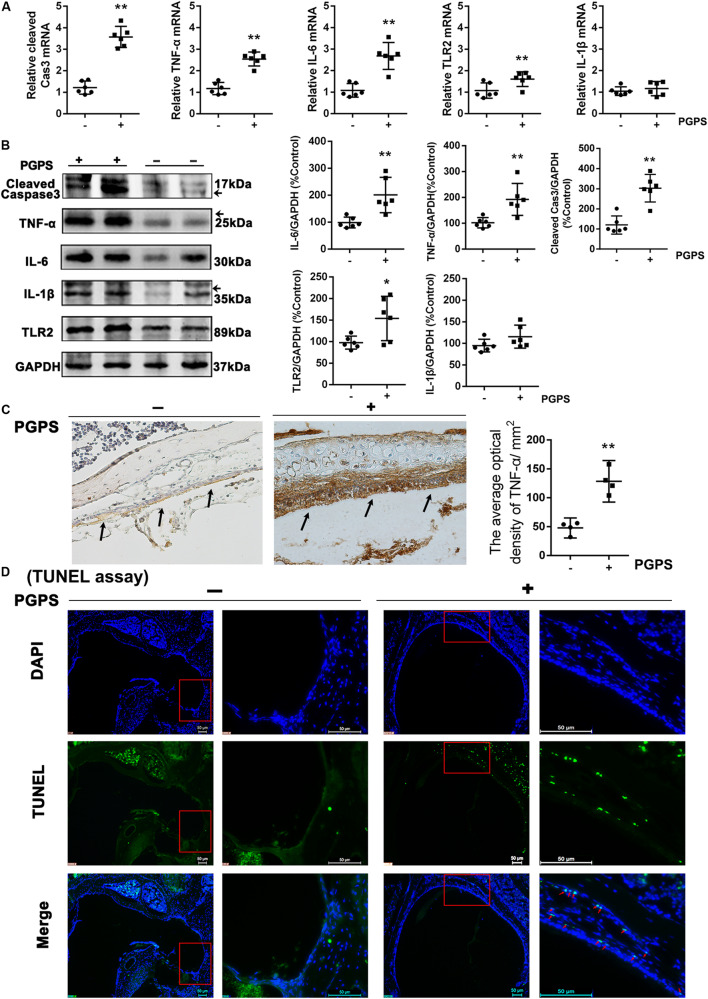
The inflammatory response and apoptosis are induced in PGPS-injected mice. **(A)** RT-PCR analysis for the transcriptional expression of caspase-3, TNF-α, IL-6, IL-1β, and TLR2 (data = mean ± 95% CI, *n* = 6 per group). **(B)** Western blot analysis for protein expression of caspase-3, TNF-α, IL-6, IL-1β, and TLR2 in the middle ear (data = mean ± 95% CI, *n* = 6 per group). **(C)** Immunohistochemistry analysis for TNF-α in the middle ear epidermal cell region of the PBS and PGPS groups. Quantitative analysis of the relative amount of TNF-α in the middle ear region (data = mean ± 95% CI, *n* = 6 per group). **(D)** A TUNEL assay was performed to assess apoptosis induction in the middle ear epidermal cell region after PGPS injection. Apoptotic cells are illustrated by the green signal in nuclei (TUNEL-positive) in the middle ear epidermal cell region. The left panels show the low-magnification view (50× magnification) and the right panels show the high-magnification view (400× magnification) for each group. The red arrows indicate TUNEL-positive cells in the PGPS group. *P < 0.05, **P < 0.01. These results confirm that the PGPS of the gram-positive bacterial cell wall is recognized by TLR2, which activates the inflammatory response and induces middle ear epidermal cell death.

To investigate the response of inflammatory molecules to PGPS-induced OM, we measured the expression of a group of inflammatory cytokines using real-time PCR and Western blot. Considering that the activation of TLR2 plays a critical role in the development of OM (Komori et al., 2011; Huang et al., 2016), we measured the transcriptional and protein levels of TLR2 and other inflammation-related genes (TNF-α, IL-6, and IL-1β). We also examined the expression of caspase-3, an important apoptotic protease. All the examined molecules were significantly upregulated in the PGPS group compared to the PBS group (Figures 2A,B). The expression of TNF-α was also assessed with IHC analysis (Figure 2C). Consistent with the Western blot results, there were only a few positive cells in the middle ear epithelium in the PBS group. In contrast, the PGPS group displayed a significant increase in the number of TNF-α-expressing middle ear epithelium cells. To further evaluate the PGPS-induced apoptosis of middle ear epidermal cells, we removed the epithelial cells from the otic bulla and performed TUNEL. This analysis revealed a significant increase in the number of TUNEL-positive middle ear epidermal cells after PGPS inoculation (Figure 2D).

### PGPS-Induced OM May Involve ER Stress

**FIGURE 3:**
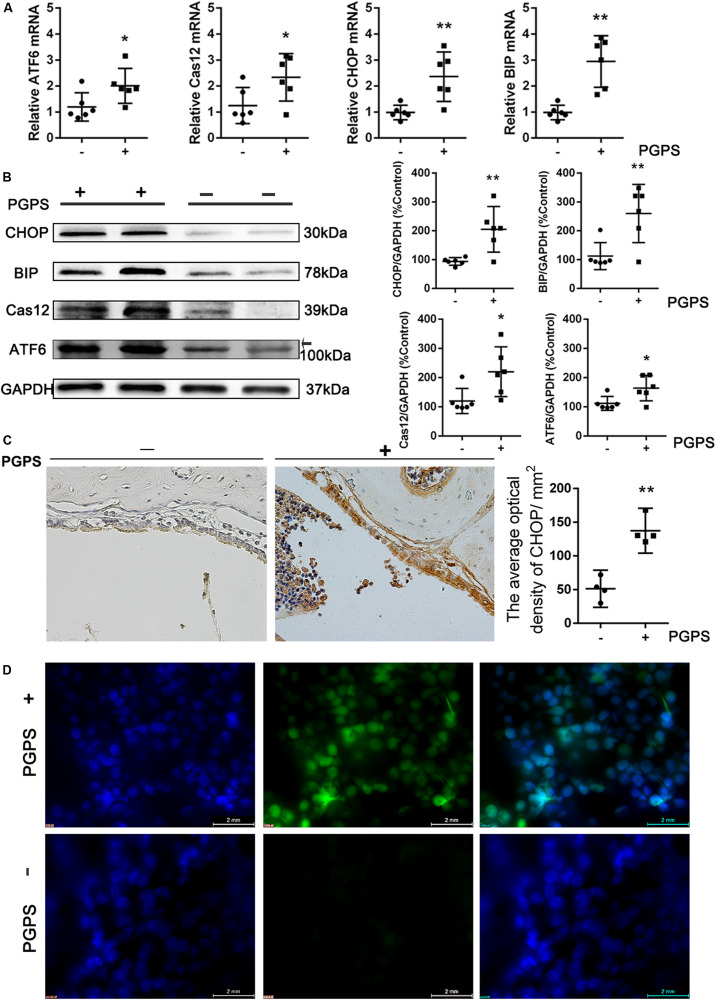
Endoplasmic reticulum stress is induced in PGPS-treated mice. **(A,B)** RT-PCR and Western blot analyses of ER stress-related molecules. ATF-6, GRP-78/BIP, CHOP, and cleaved caspase-12 levels were increased in PGPS-treated mice (data = mean ± 95% CI, *n* = 6 per group). **(C)** Immunohistochemistry analysis for CHOP. The number of cells showing CHOP immunoreactivity is significantly higher in the PGPS-treated ears than in the control ears (data = mean ± 95% CI, *n* = 6 per group). **(D)** ROS were detected by CellROX Green reagent. The green signal intensity increased in the cytoplasm and nucleus of the middle ear epidermal cells in the PGPS group. *P < 0.05, **P < 0.01.

Interaction among inflammatory responses, ER stress, and apoptosis has been reported. To determine the involvement of ER stress in PGPS-induced middle ear epidermal cell apoptosis in OM, we examined the expression of ER stress-associated cell death signaling molecules using qRT-PCR and Western blot. ATF-6, GRP-78/BIP, CHOP, and cleaved caspase-12 levels were significantly increased in the PGPS group compared to the PBS group (Figures 3A,B). The expression of CHOP was also assessed by the IHC analysis (Figure 3C). The number of CHOP-positive cells in the PGPS-induced group was significantly higher than that in the control group.

Many studies have shown that oxidative stress is involved in the pathogenic process of OM. Reactive oxygen species (ROS) can trigger ER stress, and the ROS level has been used as indirect evidence for the occurrence of ER stress. To investigate the generation of ROS, middle ear epidermis was incubated with CellROX Green reagent. The green signal intensity increased in the cytoplasm and nucleus of middle ear epidermal cells in the PGPS group. The fluorescence intensity in the nuclei was significantly higher in the PGPS group than in the PBS group (Figure 3D), indicating that PGPS enhances the production of ROS. These findings indicate that PGPS-induced OM-activated TLR2 signaling increases the expression of related inflammatory cytokines, thus perturbing ER homeostasis and amplifying the inflammatory response.

### PGPS-Induced ER Stress Plays a Key Role in the Inflammatory Response and Apoptosis in OM

**FIGURE 4:**
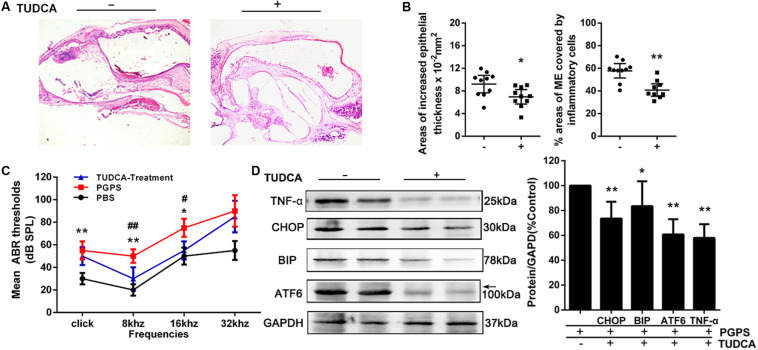
The treatment with the ER stress inhibitor, TUDCA, alleviates PGPS-induced inflammation in the middle ear. **(A)** Morphological observations of H&E-stained middle ears. TUDCA treatment reduced the inflammatory response. **(B)** The epithelial thickness and inflammatory areas in middle ear cavity. The area showing inflammatory cells is significantly larger, and the epithelial layer is significantly thicker, in the PGPS group than those in the TUDCA group (*n* = 10 per group). **(C)** ABR detection. There is a statistically significant difference in ABR thresholds between the three groups. The mean ABR thresholds in response to low-frequency (clicks and 8-kHz tone bursts) and high-frequency stimulation (16-kHz tone bursts) were significantly higher in the PGPS mice than in the PBS mice. The mean ABR thresholds in response to low-frequency (8-kHz tone bursts) and high-frequency stimulation (16-kHz tone bursts) were significantly lower in the TUDCA-treated mice than in the PGPS-injected mice (* indicates the comparison between the PGPS and the PBS group; # indicates the comparison between the PGPS and the TUDCA group; *n* = 10 per group). **(D)** Western blot analysis of TNF-α and ER stress-related proteins. ATF-6, BIP, CHOP, and TNF-α levels were decreased in TUDCA-treated mice (*n* = 6 per group). Data are presented as the mean ± 95% CI. ^#,^*P < 0.05, ^##,^**P < 0.01.

To evaluate the role of ER stress in PGPS-induced inflammation, we treated PGPS-injected mice with TUDCA, a specific ER stress inhibitor. H&E staining showed that TUDCA treatment reduced the level of middle ear inflammation (Figure 4A). The areas with inflammatory cells were significantly larger, and the epithelial layer was significantly thicker in the PGPS group than those in the TUDCA group (Figure 4B). Moreover, the increase in ABR thresholds observed in the PGPS group was prevented by TUDCA treatment at low frequencies (8 and 16 kHz), but not at 32 kHz (Figure 4C). Consistent with these functional and morphological measurements, the expression analysis revealed a reduced production of inflammatory cytokines and ER stress-related proteins (Figure 4D). These results show that TUDCA treatment can ameliorate OM. The correction of ER stress could be a potential mechanism for reduced OM.

## Discussion

Otitis media is often associated with bacterial or viral infections and, therefore, is commonly treated with antibiotics. The contributing factors of OM include genetic and environmental factors. The underlying mechanisms for the pathogenesis of OM is complicated, involving a network of signal pathways. Previous studies have revealed that the levels of TLR2 and NF-κB are upregulated in OM (Yoshimura et al., 1999; Dziarski et al., 2001; Kielian et al., 2005) and promote the recruitment of neutrophils to combat pathogens in OM (Krysko et al., 2011; Arroyo et al., 2013). These findings suggest that the TLR2 signaling pathway plays an essential role in host defense against middle ear infections. Further research is urgently needed to elucidate the inflammatory response and mechanisms of related factor interactions that could serve as intervention targets to improve the treatment of OM or to prevent the disease.

Peptidoglycan polysaccharide, a component of the gram-positive bacterial cell wall, can cause acute and chronic inflammation (Komori et al., 2011; Wu et al., 2014). Our previous study showed that PGPS could induce severe OM in TLR2-deficient (*Tlr2*^–/–^) mice (Zhang et al., 2015). In this study, we chose an optimal PGPS dose (PGPS 55 μg/10 μl) to induce OM in B6 mice to further explore the molecular mechanisms of OM. We found inflammatory infiltrates and tissue damage in the middle ear (Figure 1), thus suggesting that the OM model was successfully established. The levels of inflammation-related molecules (TNF-α, IL-6, IL-1β, caspase-3, and TLR2) were significantly upregulated (Figure 2). TLR2 enables host discrimination between self and non-self and activates the immune response to attack pathogens (Han et al., 2009). Here, PGPS was recognized by TLR2, which, in turn, activated the TLR2 signaling pathway and promoted the production of inflammatory cytokines. If the process is not controlled, inflammation occurs in the middle ear. The downstream signaling molecules, such as TNF-α, IL-6, and IL-1β, have been associated with the recurrence and persistence of OM (Mittal et al., 2014; Nokso-Koivisto et al., 2014). TNF-α is a cell-signaling protein (cytokine) involved in systemic inflammation that can be activated in the acute phase reaction. In this study, TNF-α levels were significantly increased at day 3 after PGPS injection (Figure 2), implying that acute inflammation occurred in the middle ear. IL-6, as an important mediator of the acute phase response or as an anti-inflammatory cytokine, is secreted by macrophages to stimulate the immune response during infection. IL-6 induces intracellular signaling cascades that give rise to inflammatory cytokine production in inflammatory cells, such as neutrophils (van der Poll et al., 1997; Gao et al., 2018; Farsakoglu et al., 2019). In our study, PGPS induced neutrophil infiltration (Figure 1). IL-1β, a leukocytic pyrogen, can cause a number of different autoinflammatory reactions during infection (Masters et al., 2009; Lukens et al., 2014). Our results suggest that the TNF-α, IL-6, and IL-1β play crucial roles in PGPS-induced OM. A previous study showed that TLR2 activation could cause ER stress, inflammation, and insulin resistance (Botteri et al., 2017). We hypothesized that PGPS binding to TLR2 induces ER stress to amplify inflammation in the middle ear. The expression analysis of the ER stress-related factors ATF6, BIP, CHOP, and caspase-12, supports this hypothesis (Figure 3).

ER stress is a cellular stress response related to the UPR, which involves three signaling pathways: PRKR-like ER kinase (PERK)–eukaryotic translation initiation factor 2α (eIF2α), inositol-requiring protein 1α (IRE1α)-X-box-binding protein 1 (XBP1), and ATF6. ATF6 is a basic leucine zipper transcription factor. Under stress, the pro-protein (approximately 90 kDa) translocates to the Golgi, where it is cleaved by proteases to form an active transcription factor (approximately 50 kDa) in the UPR. In this study, the ATF6 level was increased in PGPS-induced OM (Figure 3), indicating ER stress activation by ATF6 signaling pathways. Growing evidence suggests that ATF6 activation occurs rapidly, but its attenuation requires time. In this study, we hypothesized that the protein expression of ATF6 was increased by the upregulated expression of TLR2. The molecules BIP and CHOP, downstream of ATF6, are targets of the ER stress response and can effectively activate UPR pathways during ER stress (Chapman et al., 1998; Okamura et al., 2000). Based on our analysis, the expression levels of BIP and CHOP were significantly increased in the PGPS-induced group (Figure 3), indicating that the ER stress response is activated in OM.

Research has suggested that aberrant accumulation of ROS can induce ER stress (Volmer et al., 2013). CHOP can activate ER oxidase 1α (ERO1α) to augment the production of ROS. Thus, ER stress can induce oxidative stress, which leads to cell death. Alternatively, loss of adaptation to ER stress (Guan et al., 2017) can also increase proinflammatory gene expression via activation of NF-kB (Schmitz et al., 2018). Both of these mechanisms can contribute to the inflammatory process in OM (Aydogan et al., 2013; Balikci et al., 2014). This is consistent with previous research, which found that ER stress, inflammation, and oxidative stress are tightly integrated and each one can influence the other (Zhang et al., 2017; Chen et al., 2019; Diaz-Bulnes et al., 2019). The inflammatory response can be activated by maladaptive ER stress conditions, and inflammatory gene expression and disturbed redox homeostasis in the ER can further enhance ER stress (Zhang et al., 2017). In this study, our CellROX Green reagent staining revealed that ROS levels were increased (Figure 3D). Therefore, PGPS associates with increased ROS-positive cells, increased ER stress markers, and increased inflammation. Although our data cannot conclude if the increased ROS levels is the result of ER stress or the result of the PGPS-mediated responses, our data clearly show that TUDCA-mediated improvement of protein folding in the ER decreases proinflammatory gene expression. Increased ROS levels are expected to oxidize free cysteines in the ER and/or cause mixed disulfide bond formations and protein misfolding (Gao et al., 2020). Therefore, independently if ROS proceeds or follows ER stress, it is expected to increase protein misfolding in the ER, decrease adaptive responses, and increase proinflammatory programs. Our data suggest that PGPS-mediated induction of ER stress contributes to the amplitude of the inflammatory response. Rescuing ER stress can delay proinflammatory gene expression and PGPS-induced OM (Figure 4).

## Conclusion

In conclusion, our findings show that PGPS-induced OM-related ER stress plays a key role in inflammation in the middle ear. Therefore, the interaction between PGPS-induced TLR2 signaling and ER stress in OM warrants further exploration. Additionally, this study indicates that a signaling crosstalk occurs between ER stress and oxidative stress in OM, signifying the need for further studies. Together, our study raises the possibility that the inhibition of ER stress could serve as a potential therapeutic approach for the prevention of OM.

## Data Availability Statement

All datasets generated for this study are included in the article/supplementary material.

## Ethics Statement

The experimental protocol was approved by the Animal Use and Care Committee of the Binzhou Medical University Hospital. Studies were conducted according to the principles set forth in the Guide for the Care and Use of Laboratory Animals (DC005846) as well as the Institute of Laboratory Animal Resources (protocol 2014-0155).

## Author Contributions

XZ, ToZ, and XL performed the experiments. HZ, XZ, BL, and QZ wrote the manuscript. JuaC analyzed the data. YW and JuC participated in the discussion of the project. QZ, XZ, and ZF designed the study. BL, QZ, XZ, and MH acquired funding. XZ, QZ, TiZ, BH, and MH revised the manuscript. All authors reviewed and approved the manuscript.

## Conflict of Interest

The authors declare that the research was conducted in the absence of any commercial or financial relationships that could be construed as a potential conflict of interest.
